# Eine explorative Untersuchung der Einflüsse von ABW-Büromerkmalen auf die Wahrnehmung der Organisationskultur

**DOI:** 10.1007/s11612-022-00631-y

**Published:** 2022-04-19

**Authors:** Clara Weber, Pascale Bébié-Gut, Alyssa Riebli, Lukas Windlinger

**Affiliations:** Department of Life Sciences and Facility Management, Zurich University of Applied Sciences, Einsiedlerstrasse 2, 8820 Wädenswil, Schweiz

**Keywords:** Organisationskultur, Bürogestaltung, Aktivitätsorientierte Arbeitsumgebungen, Competing Values Framework, Workplace Management, Organizational culture, Office layout, Activity based working, Competing values framework, Workplace management

## Abstract

Dieser Beitrag der Zeitschrift Gruppe. Interaktion. Organisation. (GIO) untersucht, wie Designmerkmale aktivitätsorientierter Arbeitsumgebungen (activity-based working, ABW) Einfluss auf die Wahrnehmung von Clan-Organisationskulturen nehmen können. Bisherige Forschungsarbeiten haben Designmerkmale selten isoliert von anderen Eigenschaften der physischen Umgebung betrachtet und in Zusammenhang mit Organisationskulturen gestellt. Es wurden halbstrukturierte Interviews mit acht Teilnehmenden aus verschiedenen Organisationen geführt, die eine ABW-Umgestaltung mit Ziel einer hierarchisch flachen Clan-Kulturentwicklung miterlebt haben. Die Mehrheit zuvor berichteter *funktionaler* und *assoziativer* Einflüsse auf Clan-Kultur-Indikatoren wie *Kommunikation, Zusammenarbeit, Beziehungen, Zugehörigkeit* und *kollegial-beratende Führungskultur* wurden identifiziert. Besonders Unterthemen, wie z. B. *dynamische Atmosphäre, fokussierter Austausch, Offenheit* und *Firmenidentifikation* wurden bestätigt oder neu identifiziert. Querschnittsthemen, die funktionale und assoziative Einflussmechanismen vertiefen, waren *Zugänglichkeit, Serendipität, psychologische Distanz *und* Territorialprinzipien*. Die Ergebnisse deuten darauf hin, dass die Bürogestaltungsmerkmale auf komplexe Weise mit der Organisationskultur zusammenhängen; die Bürogestaltung scheint die Organisationskultur unterstützen zu können.

## Abstract (en)

This qualitative study sets out to explore *how* three office design features, that are typically found in activity-based working (ABW) environments, influence employees’ perception of organizational culture. Overall, little is known about how design features of new office concepts, such as ABW, reflect and reinforce an intended hierarchy-flat organisational culture. Semi-structured interviews were conducted with eight participants of different organisations who moved to an ABW environment with the intend to foster a hierarchy-flat clan-culture. The transcripts were subjected to thematic analysis with a semi-deductive approach. Results on *how* design features were perceived to influence organisational culture were categorised into *functional* and *associative* mechanisms. Majority of previously found *functional* and *associative* influences for each of the three design features were validated; influences on clan-culture indicators such as *communication, collaboration, relationships, group cohesion, and collegial leadership style* were demonstrated. Particularly sub-themes such as *dynamic atmosphere, focused information flow, openness attitude and organisational identification* were found or newly identified Transcending themes, which add to an in-depth evaluation of functional and associative influences include *accessibility, serendipity, psychological distance* and *territoriality*. Findings suggest that perceptions of office design features are associated with employees’ perceptions of the organizational culture and employee attitudes in complex ways; office design appears to be useful to support organisational culture and to reflect culture principles in spatial form.

## Einführung: Bürogestaltung als Vermittler

Obwohl Einflüsse der Bürogestaltung auf Nutzerverhalten im Allgemeinen (z. B. Windlinger et al. 2014) sowie auf Organisationskultur im Speziellen (z. B. Hong et al. 2006; McElroy und Morrow 2010; Zerella et al. 2017) bekannt sind, haben nur wenige Studien spezifische Designmerkmale und deren Einfluss auf bestimmte Typen von Unternehmenskultur untersucht (vgl. Zerella et al. 2017). Ebenso fokussierten sich ein Grossteil bisheriger Studien auf den Vergleich von Bürolayouts (offen vs. geschlossen), anstatt spezifische Bürokonzepte mit einer angestrebten Organisationskultur in Zusammenhang zu bringen. Aufgrund des Mangels an fokussierter Forschung, lag der primäre Fokus neuerer Studien auf korrelativen Zusammenhängen spezifischer Designmerkmale und angestrebter gesamtheitlicher Organisationskultur (Zerella et al. 2017). Ausstehend sind weitere Evidenzen, die diesen Zusammenhang bestätigen, sowie tiefgreifende Analysen, *wie* Designmerkmale Einfluss auf Organisationskulturen nehmen können. Angesichts zunehmender Flexibilitätsanforderungen an Organisationen und deren Bürokonzepte (Brill et al. 2001; Hartnell et al. 2011; Windlinger et al. 2015) ist dieses Forschungsthema von besonderer Relevanz. In diesem Beitrag wird dargelegt, inwiefern spezifische Designmerkmale aktivitätsbasierter Büroumgebungen (ABW) eine angestrebte Organisationskulturveränderung in acht Fällen unterstützte.

### Framework der Organisationskultur & Clankultur

Die Organisationskultur umfasst grundlegende Annahmen, Werte, Normen und Praktiken die im Unternehmen akzeptiert oder gefördert werden (Schein 2004). Kulturtypen werden in Studien häufig mittels des Competing Value Framework (CVF) differenziert (Howard 1998). Insbesondere unterscheidet die Empirie (Cameron und Quinn 2011) zwischen vier Kulturformen: Clan, Hierarchie, Market und Adhokratie.

Die Clankultur (auch Konsenskultur) ist als „Human Relations Modell“ zu verstehen, in dem zwischenmenschliche Beziehungen und Arbeitsflexibilität im Vordergrund stehen (Nanayakkara und Wilkinson 2021, S. 137). Zusammenarbeit zwischen Angestellten ist die Hauptarbeitsausrichtung, während Führungskräfte als vermittelnde oder beratende Person agieren (Denison et al. 2004). Schwerpunkte liegen u. a. auf Zusammenhalt, Zugehörigkeit, Unterstützung, offener Kommunikation, Zusammenarbeit, guten Beziehungen und nicht-hierarchischem Verhalten (Hartnell et al. 2011). Örtlich und zeitlich flexible Arbeitsformen oder flexible Arbeitsplatzkonzepte, wie aktivitätsorientierte Arbeitsumgebungen (activity-based working, ABW), sind in diesem Kulturtyp oft vertreten, da sie Flexibilität und Autonomie bieten (Nanayakkara und Wilkinson 2021). Insbesondere das Arbeitsplatzkonzept ABW unterstützt verschiedene flexible Formen der Arbeitsausführung und ist hierarchisch flach (z. B. haben auch Führungskräfte meist kein eigenes Büro). Daher ist es naheliegend, dass für den Clankulturtyp flexible Arbeitsplatzkonzepte, wie ABW, als am besten passend postuliert wurden (Hartnell et al. 2011; Nanayakkara und Wilkinson 2021; Zerella et al. 2017).

### Aktivitätsorientierte Arbeitsumgebungen & ihre Designmerkmale

Das Hauptprinzip aktivitätsorientierter Arbeitsumgebung (ABW) ist die Bereitstellung verschiedener Arten von Arbeitsplätzen auf einer Fläche, die jeweils eine bestimmte Art von Arbeitstätigkeiten unterstützen (Fuchs et al. 2018; Windlinger et al. 2015). Hinsichtlich der offenen Gestaltung zeigt das ABW Ähnlichkeiten zum Grossraumbüro, bietet aber eine Vielfalt an offene und geschlossenen Arbeitsmöglichkeiten. Arbeitsplätze sind in der Regel nicht zugewiesen, Teams sind aber mehrheitlich in Zonen zusammengeführt. Prinzipiell sollen Nutzendewählen, wo und in welchem Umfeld sie arbeiten möchten; somit soll eine optimale Passung zwischen Arbeitsumgebung und Arbeitsaktivität erreicht werden (Fuchs et al. 2018; Windlinger et al. 2015). Designmerkmale, welche Kernprinzipien des ABWs repräsentieren, sind *visueller Zugang, räumliche Nähe* und *Arbeitsplatzgleichheit*.

### Designmerkmale & Organisationskultur

Bestehende Evidenzen zum Zusammenhang zwischen Designmerkmalen und Organisationskultur werden folgend anhand eines Frameworks zu Einflussmechanismen der physischen Büroumgebung entlang von zwei der drei von Vilnai-Yavetz et al. (2005) vorgeschlagenen Dimensionen vorgestellt. 1) *Funktionalität* betrifft die Unterstützung oder Hinderung der Umwelt in der Erreichung von Arbeitszielen. 2) *Symbolik *umfasst raumbezogene Assoziationen, welche die Bedeutung und Interpretation der gebauten Umwelt vertiefend erschliessen sollen. Zur Vereinfachung wird der Begriff *Assoziationen* verwendet. 3) *Ästhetik* betrifft ästhetische Evaluationen und wurde für die vorliegende Studie ausgeschlossen, da es keine Evidenzen für einen Zusammenhang zu Kulturparametern gab.^1^^,^^2^

#### Visueller Zugang

Visueller Zugang umfasst, andere sehen zu können, ohne den Arbeitsplatz zu verlassen und wird durch physische Barrieren im Raum bestimmt (Becker und Sims 2001).

##### Funktionale Einflüsse

Studien, die verschiedene offene (Multispace, Open Plan, ABW) und geschlossene Büroformen (Zellenbüros; Cubicles) miteinander verglichen und sich direkt oder indirekt (Offenheit) auf visuellen Zugang bezogen, beobachteten vor allem Veränderungen in der Kommunikation (Boutellier et al. 2008) sowohl im Team (Becker und Sims 2001; De Been et al. 2015; Stryker 2004) als auch teamübergreifend (Allen und Gerstberger 1973; De Been et al. 2015; Stryker 2004). Die Veränderungen betrafen die Häufigkeit (Allen und Gerstberger 1973; Becker und Sims 2001; Boutellier et al. 2008; Stryker 2004) sowie die Art der Kommunikation; besonders in ABW-artigen Büros wurde, aufgrund der Offenheit, über verkürzte Kommunikationsdauer, schnelleren und informelleren Informationsaustausch (Boutellier et al. 2008; Becker und Sims 2001), schnellerem Fällen von Entscheidungen (Becker und Sims 2001) und mehr Wissensaustausch (De Been et al. 2015) berichtet. Des Weiteren wurde visueller Zugang mit veränderter Führungskultur hinsichtlich hierarchie-übergreifender Kommunikation, verbesserten Beziehungen und Coachingfunktion in Verbindung gebracht (Becker und Sims 2001); jedoch besteht hier eine Überschneidung zur Arbeitsplatzgleichheit (siehe 4.3). Unklar zuzuordnen war das Phänomen der zufälligen Begegnungen (Serendipität), welche das Kennenlernen neuer Kolleginnen und Kollegen, Kommunikation (De Been et al. 2015; Boutellier et al. 2008) und eine dynamische Atmosphäre (De Been et al. 2015) begünstigte; einige Autorinnen und Autoren brachten Serendipität in Zusammenhang mit Offenheit des Layouts (De Been et al. 2015) andere mit räumlicher Nähe (Kiesler und Cummings 2002).

##### Assoziative Einflüsse

Studien, die offene Bürostrukturen mit geschlossenen verglichen, legen nahe, dass durch erhöhte Sichtbarkeit Konflikte durch Erkennung von non-verbaler Hinweisen (Mimik und Gestik) vermieden werden konnten (Becker und Sims 2001). In einer ABW-spezifischen qualitativen Untersuchung wurden Zusammenhänge zwischen visuellem Zugang und teamübergreifendem Zusammenhalt dargelegt und von einer teamübergreifenden Angleichung sozialer Normen berichtet (Skogland 2017).

#### Räumliche Nähe

Räumliche Nähe betrifft die physische Distanz zwischen Mitarbeitenden auf einer Bürofläche (Kiesler und Cummings 2002).

##### Funktionale Einflüsse

In vergleichenden Studien von Zellen- und Grossraumbüros wurde steigende Kommunikationshäufigkeit, mehr Kommunikationspartner (Allen und Gerstberger 1973; Stryker 2004), ein verbesserter Informationsfluss (Hong et al. 2006) und Zusammenarbeit hinsichtlich Koordination und Planung (Allen und Gerstberger 1973) festgestellt, was in der Ergebnisdiskussion mehrheitlich mit räumlicher Nähe und Zugänglichkeit erklärt wurde. In traditionellen Settings waren Unterhaltungen (Allen und Gerstberger 1973; Kraut et al. 1990; Zahn 1991) und Kollaboration (Kraut et al. 1990) mehrheitlich auf Büronachbarinnen oder Büronachbarn beschränkt. Kiesler und Cummings (2002) postulieren in ihrem Review, dass ausreichend Evidenzen bestünden, dass Nähe (von Büros) zufällige Begegnungen (Serendipität) begünstigt (siehe auch 4.1).

##### Assoziative Einflüsse

In Studien, die klassische Büroformen untersuchten, wurde festgehalten, dass durch räumliche Nähe soziale Beziehungen und Zusammenhalt intensiviert werden, da besonders der informelle Austausch wahrscheinlicher wird, man sich besser kennenlernt und die Vertrautheit steigt (Kraut et al. 1990; vgl. Kiesler und Cummings 2002). Weiter leiten Kiesler und Cummings (2002) her, dass physische Nähe und geteilte Territorien Teamzugehörigkeit und -Identifikation fördern (auch Elsbach und Bechky 2007).

#### Arbeitsplatzgleichheit

Arbeitsplatzgleichheit bezieht sich auf Ähnlichkeiten der Arbeitsplätze von Angestellten und Vorgesetzten (Zerella et al. 2017).

##### Funktionale Einflüsse

Studien, die geschlossene mit offenen Büroumgebung im Längs- oder Querschnitt verglichen und auf Arbeitsplatzgleichheit verwiesen, berichteten von clan-verwandten Verhaltensveränderungen. Dazu zählen allgemein informellere und kooperativere Kultur (McElroy und Morrow 2010), hierarchie-flachere Kultur (Skogland 2017), mehr Interaktion und hierarchie-übergreifende Besprechungen (Hong et al. 2006; Becker und Sims 2001), hierarchie-übergreifende Beziehungen und kollegial-beratender Führungsstil (Becker und Sims 2001). Inwiefern diese Veränderungen jedoch explizit mit Arbeitsplatzgleichheit in Zusammenhang standen, ist unklar.

##### Assoziative Einflüsse

Verschiedene Studien zeigen, dass Räumlichkeiten zur Statusrepräsentation genutzt werden (Hirst 2011) und diese Hierarchien untermauert (Elsbach und Bechky 2007; Zalesny und Farace 1987; Zhang und Spicer 2014); ebenso ist bekannt, dass Status beeinflusst, wie über Hierarchiestufen hinweg kommuniziert und interagiert wird (Cameron und Quinn 2011). Jedoch liegt der Fokus empirischer Studien mehrheitlich auf Arbeitsplatzungleichheiten, Status und hierarchischer Führungskultur (Zhang und Spicer 2014). Insofern ist der theoretisch postulierte Zusammenhang, zwischen einer hierarchie-flachen Führungskultur, unterstützt durch räumlich symbolisierte Angleichung des Status und verringerter psychologischer Distanz (Becker und Sims 2001), und Clan-Kulturindikatoren, wie flache Hierarchien, Zusammenarbeit und Beziehungsförderung (Elsbach und Bechky 2007) noch nicht vollständig belegt.

### Forschungsfrage

*Wie nehmen die ABW Designmerkmale visueller Zugang, räumliche Nähe und Arbeitsplatzgleichheit Einfluss auf die Wahrnehmung von Clan-Kultureigenschaften?*

- Können bekannte Zusammenhänge anderer Studienkontexte auf ABW und Clan-Kultur übertragen werden?
- Können die durch Vorstudien identifizierten Zusammenhänge zwischen ABW Designmerkmalen und Clan-Kultureigenschaften qualitativ bestätigt werden?
- Können neue Zusammenhänge identifiziert werden?

## Methode

Ein qualitatives narratives Verfahren wurde gewählt, um funktionale sowie assoziative Zusammenhänge zwischen Designmerkmalen und Organisationskultur explorativ zu untersuchen.

### Forschungsdesign, Datenerhebung und Analyse

Die Studie umfasst halb-standardisierte leitfadengestützte Interviews (*N* = 8) in Unternehmen verschiedener Branchen und berücksichtigte Beschäftigte sowie Personen mit Personal- und Geschäftsverantwortung. Mehrere Unternehmen, Branchen und Arbeitspositionen einzubeziehen, sollte eine hohe Varianz an Erfahrungswerten zur Arbeitskultur erzielen. Einschlusskriterien für die Interviewteilnahme waren: Miterleben einer ABW-spezifischen Büroumgestaltung; Mindestarbeitserfahrung im aktivitätsbasierten Arbeitsumfeld von sechs Monaten (gemäss Mindestanstellungsdauer in Vergleichsstudien, z. B., McElroy und Morrow 2010); Clan-Organisationskultur in Arbeitsgruppe/Abteilung/Unternehmen bestehend oder in Entwicklung. Rekrutierungsemails wurden an Unternehmen des Forschungsnetzwerkes versendet. Emails enthielten Definitionen zu ABW und Clan-Organisationskultur. Interessierte Teilnehmende stuften sich demnach selbst als zulässig/geeignet ein, was anschliessend durch ein Telefonat verifiziert wurde. Eine Mindestanzahl von *N* = 12 Teilnehmenden wurde angestrebt; eine Orientierungsgrösse (Fusch und Ness 2015), die sich aus vorherigen qualitativen Studien zu diesem Thema ergibt (z. B., Klaffke und Kuchta 2015). Aufgrund der COVID-19 Pandemie und der damit verbundenen Anforderungen, mussten fünf potenzielle Teilnehmende das Interview absagen.

Die ersten fünf Interviews wurden in der Arbeitsumgebung der Teilnehmenden durchgeführt; die letzten drei fanden, aufgrund von Pandemie-Sicherheitsmassnahmen, per Videoanruf statt. Tonaufnahmen wurden wortgetreu transkribiert.

Die Auswertung der Transkripte erfolgte semi-deduktiv (Fereday und Muir-Cochrane 2006) mittels thematischer Analyse (Braun und Clarke 2006). Das semi-deduktive Verfahren kombiniert den datenorientierten induktiven mit dem vorstrukturierten deduktiven Verfahren mittels Vordefinition von Hauptthemen (visueller Zugang, räumliche Nähe, Arbeitsplatzgleichheit) und Unterthemen (funktional und assoziativ; siehe Tab. 1, Kategorie K; Crabtree und Miller 1999). Der Kodierungsprozess folgte dem sechsstufigen thematischen Analyseverfahren nach Braun und Clark (2006), welches in jeder Stufe eine iterativ reflexive Analyse innerhalb der Forschungsgruppe voraussetzt. Die ersten beiden Stufen (Einarbeiten und Kodieren) erfolgten induktiv; Codes wurden als angemessen erachtet, wenn sie folgendem Kriterium entsprachen: [they capture] „the qualitative richness of the phenomenon“ under investigation (Fereday und Muir-Cochrane 2006, S. 83). Anschliessend wurden die einzelnen Codes durch einen deduktiven theoriegeleiteten Ansatz präzisiert (Crabtree und Miller 1999). Die dritte Stufe (Suche nach Hauptthemen) erfolgte mittels deduktivem Scan und induktiver Anpassung in drei Unterschritten: Gruppierung der Codes in drei vordefinierten Haupt- und Unterthemen (Tab. 1, Kategorie R); Ergänzung von drei weiteren Hauptthemen (Querschnittsthema – Q, Beeinflusste Organisationskulturparameter – K, Zusammenführendes Thema Haupteinfluss Organisationskultur – Z; siehe Tab. 1) gemäss induktivem Ansatz; Gruppierung der Codes in Unterthemen innerhalb der Hauptthemen. Die vierte und fünfte Stufe (Überprüfung und finale Betitelung) erfolgte semi-deduktiv; das heisst es erfolgte eine fortlaufende iterative Analyse der Themen mit Rückbezug auf die Literatur. Die Forschungsgruppe befand, dass Datensaturierung erreicht wurde (keine neuen Codes und Themen; Daten erschienen „rich“ und „thick“, Fusch Ness, S. 1409). Schritt sechs umfasst die Verschriftlichung der Methode und Ergebnisse (siehe auch Tab. 1).

**Table 1:** 

Designmerkmal	Art des Einflusses	Thema Räumliche Spezifierung (R)	Querschnittsthema Vertiefender assoziativer/funktionaler Mechanismus (Q)	Thema Beeinflusste Organisationskulturparameter (K)	Zusammenführendes Thema Haupteinfluss Organisationskultur (Z)
*Visueller Zugang*	Funktional	1. Räumliche Barrierefreiheit Sichtachsen	Zugang zu Teammitgliedern	1.1 Häufigkeit des Austausches: Mehr formeller und informeller Austausch	Kommunikation (häufig, schnell, informell und spontan)
				1.2 Art des Austausches i. Kürzer, schneller, spontaner (im Team) ii. Verschlankung/Fokussierung Inhalt (im Team) iii. Informellerer, unbürokratischer (zwischen Teams)	
	Assoziativ	1. Sichtachsen	Territorialprinzipien	1.1 Dynamische Atmosphäre	Dynamische Atmosphäre Zugehörigkeit
		2. Überblick		1.2 Zugehörigkeit	
*Räumliche Nähe*	Funktional	1. Räumliche Zusammenführung eines Teams	Zugänglichkeit Teammitglieder	1.1 Im Team Zusammenarbeit: Einfachere Koordination Planung	Beziehungen zwischen Teams Zusammenarbeit in und zwischen Teams
				1.2 Im Team Zusammenarbeit: Mehr Produktivität	
				1.3 Im Team Zusammenarbeit: Schneller Informationswechsel	
		2. Räumliche Nähe anderer Teams	Serendipität	2.1 Teamübergreifend mehr Zusammenarbeit	
				2.2 Teamübergreifend informierter über Arbeit anderer	
				2.3 Teamübergreifend neue und verbesserte Beziehungen	
	Assoziativ	1. Räumliche Zusammenführung eines Teams	Territorialprinzipien	1.4 Teamzugehörigkeit	Zugehörigkeit in zwischen Teams
				1.5 Soziale Normen im Team	
		2. Räumliche Zusammenführung mehrerer Teams	Territorialprinzipien	1.1 Teamübergreifende Offenheit	
				1.2 Teamübergreifende Zugehörigkeit	
				1.3 Teamübergreifend Identifikation	
*Arbeitsplatzgleichheit*	Assoziativ	1. Angleichung der Arbeitsbedingungen	Territorialprinzipien verringerte psychologische Distanz	1. Symbolische Angleichung des Status/flache Hierarchie	Kollegial-beratende Führungsbeziehung
		2. Teilung einer Zone		1. Präsenz der Führungskraft im Team	
				2. Gefühlte Nähe zu Führungsperson	
				3. Transparenz im zwischenmenschlichen Umgang	

### Interviewteilnehmende

Die Erhebung fand, zwischen Februar und März 2020, in mittelständischen Unternehmen der Branchen Gesundheit (3), halb-öffentlicher Bereich (4) und Beratung (1) in der Schweiz statt. Die willkürliche Stichprobe bestand aus Teilnehmenden zwischen 25 und 50 Jahren (*M* = 38, *SD* = 8,93), hauptsächlich männlichen Geschlechts (sieben Männer), fast zur Hälfte aus dem mittleren Management (3), die seit durchschnittlich 8 Jahren (*SD* = 8,53) im Unternehmen tätig waren und durchschnittlich 3 Jahren (*SD* = 1,69) in einem ABW-Büro arbeiteten. Teilnehmende waren in den Abteilungen Engineering (3), Facility Management (3) und IT (2) tätig. Die Teilnehmenden waren auskunftsfähig, da sie alle eine ABW-spezifische Büroumgestaltung vor durchschnittlich 3 Jahren (*SD* = 1,69; Bandbreite 1–7 Jahre) miterlebt haben und im Unternehmen, Abteilungen oder Arbeitsgruppen eine Clan-Organisationskultur bestehend war oder sich in Entwicklung befand.

## Ergebnisse

Wie in Tab. 1 dargestellt, wurden folgende Themen identifiziert, welche im Text mit Kürzeln gekennzeichnet sind: Räumliche Spezifizierungsthemen des Designmerkmals (R), vertiefende Querschnittsthemen zu funktionalen oder assoziativen Mechanismen (Q), beeinflusste Kulturparameter (K), sowie zusammenfassender Kultureinfluss (Z).

### Visueller Zugang

Kulturveränderungen durch visuellen Zugang wurden *funktionalen* und *assoziativen Mechanismen *zugeordnet.

#### Funktional: Kommunikation schnell spontan durch Barrierefreiheit Sichtachsen

Es wurde berichtet, dass durch die Aufhebung von räumlichen Barrieren und der Erstellung von Sichtachsen (R 1), der Zugang zu Teammitgliedern (Q) erleichtert wurde. Interviewteilnehmende berichteten, dass dies die *Häufigkeit *(K 1) sowie *die Art des Austausches *(K 2) zwischen Kollegen drastisch veränderte. Ebenso wurde berichtet, dass der *formelle und informelle* Austausch häufiger wurde. Des Weiteren nahmen einige Teilnehmende eine Veränderung des Austausches innerhalb des Teams war. Dieser wurde *kürzer, schneller und spontaner* wahrgenommen und durch die Regelmässigkeit, inhaltlich *fokussierter*:

Viel mehr Austausch, weil man sich auf der Fläche auch sieht. Sonst musste man auch vielleicht ein Meeting organisieren und dann spricht man dann wirklich eher die Traktandenliste durch und jetzt hat man auch für privates Nachfragen (…) mal Zeit zwei/drei Minuten (Nr. 4).

Mehr Besprechungen aber kürzere, spontane und just in time-Meetings. Die Qualität ist wahrscheinlich besser geworden (Nr. 7).

Jetzt gehst du schon eher an den Tisch heran und fragst, hey hast du kurz Zeit, können wirs kurz

anschauen? Von daher ist der Umgang schon einfacher geworden. (Nr. 9).

Aus den anderen Gruppen, die waren einfach verfügbar. Man konnte einfach hingehen und kurz eine Frage stellen, die war gleich geklärt. Man musste nicht lange Mails schreiben (Nr. 4).

Fokussierter (…) gerade wenn man sich häufiger auch sieht (…) Dann kommt man auch schneller (…) auf den Punkt (Nr. 5).

Die Schilderungen der Teilnehmenden deuten darauf hin, dass eine Aufhebung von räumlichen Barrieren und die Erstellung von Sichtachsen, eine *Kommunikationsform* fördern könnte, die *häufig, schnell, informell und spontan* (Z) ist.

#### Assoziativ: Dynamik Zugehörigkeit durch Sichtachsen Überblick

Die Aussagen der Teilnehmenden geben Hinweise darauf, dass die Erstellung von Sichtachsen (R 1) nicht nur zur einer schnellen und spontanen Kommunikation beigetragen hat; die Teilnehmenden empfanden auch eine Veränderung hinsichtlich der Arbeitsatmosphäre. Diese wurde als *dynamischer* (K 1) wahrgenommen und war geprägt durch *Schnelligkeit und Spontanität.* Einige berichteten, dass die Verschaffung des Überblicks (R 2) über die territorial-geteilte Teamzone (Q), ein Gefühl der *Zugehörigkeit *(K 2) mit sich brachte:

Die Offenheit des Raumkonzepts hier lässt zu, dass man in mittleren Gruppen schnell zusammenkommt oder die Leute zusammenführt (Nr. 5).

Für die Sitzung und für mich ist das [ABW] super, weil ich kann mich schnell mit den Leuten … treffen, ohne grosse Arbeitszeiten. Für mich ist das super (Nr. 1).

Ich glaube der Austausch ist intensiver […], dass man sich häufiger sieht, dass man schneller … zu einem Meeting abmachen kann, wo die Leute Zeit haben (Nr. 5).

Du gehst eher einmal unangekündigt zu deinem Teamkollegen und sagst, hey können wir das kurz anschauen? (Nr. 9).

Übersichtlich, sieht man eigentlich jeden Morgen alle Leute und begrüsst sie auch (Nr. 3).

Die Schilderungen der Teilnehmenden deuten darauf hin, dass die Erstellung von Sichtachsen und territorialem Überblick, eine dynamische Atmosphäre und ein Gefühl der Zugehörigkeit (Z) fördern könnte.

### Räumliche Nähe

Kulturveränderungen durch räumliche Nähe wurden *funktionalen* und *assoziativen Mechanismen *zugeordnet.

#### Funktional: Zusammenarbeit Beziehungen durch räumliche Zusammenführung Serendipität

Teilnehmende berichteten, dass sie durch die räumliche Zusammenführung ihres Teams (R 1), erleichterten Zugang zu ihren Teammitgliedern (Q1) hatten. Den Ausführungen zufolge, brachte dies eine Erleichterung oder Verbesserung in *Koordination und Planung* (K 1), *Informationsaustausch* (K 3) und *Produktivität in der Teamarbeit* (K 2) mit sich:

Das machts für uns einfach viel einfacher [zu planen], weil wir relativ viel zusammenarbeiten, zusammen etwas besprechen müssen. Da macht es Sinn, dass wir in Sichtnähe hocken (Nr. 9).

Jetzt eben alle in einer Fläche (…) dann schreibt man keine Mail, da geht man einfach kurz hin und fragt. (…) besonders für die Leute, die viel von anderen wissen müssen, ist das ein sehr grosser Vorteil und für mich selbst auch, denn es geht schneller (Nr. 4).

Das macht ja vielleicht auch Sinn, wenn alle Leute beieinander sind und so einfacher kommuniziert werden kann (Nr. 9)

Austausch ist intensiver, vor allem der Face-to-Face Austausch. Eben dadurch bedingt, dass die Leute auch näher zusammengerückt sind. (…) habe als Ganzes das Gefühl, dass es durchaus produktiver geworden ist, eben durch einen intensiveren Austausch (Nr. 5).

Die Qualität ist wahrscheinlich besser geworden. […] Das ist natürlich schon besser geworden, weil wir natürlich auf einem Geschoss mehrere Teams sind vom gleichen Bereich. Das hat sich natürlich schon sehr sehr sehr verbessert (Nr. 7).

Ebenso berichteten Teilnehmende, dass die räumliche Nähe anderer Teams (R 2), Serendipität (zufällige Begegnungen, Q 2). Einige empfanden, dass dies *teamübergreifende Zusammenarbeit, Austausch *und* Beziehungen *(K 1–3) förderte:

Also ich zumindest habe vorhin nicht über die Gruppen, also über das X‑Team [anonymisiert] hinaus Kooperationen mit anderen gehabt. Das hat sich dann, würde ich meinen auch wegen der veränderten Büroumgebung verändert (Nr. 4).

Plötzliches Treffen (…) vermischen sich die Teams cross-funktional und arbeiten an verschiedenen Themen (Nr. 1).

Früher (…) die Leute teils gar nicht gekannt hat. Also den Namen gehört und ein Foto dazu und hat die Menschen nicht gekannt (…) jetzt (…) sich begegnet und einfach auch mal darüber spricht: Hey, was machst du gerade und dann merkt man, man hat mega viel Gemeinsamkeiten in Projekten oder auch Privat (Nr. 8)

Viele Personen, mit denen man vorher noch nicht so zu tun hatte, trifft man plötzlich. Sei es im Kaffeeraum oder eben auf dem Gang oder so. Es gibt schon eine grössere Durchmischung und das gibt positive Gespräche (Nr. 6).

Die Schilderungen der Teilnehmenden deuten darauf hin, dass ein erleichterter Zugang zu Teammitgliedern (Q 1) sowie Serendipität (Q 2), forciert durch räumliche Nähe andere Teams (K 2), die Teamzusammenarbeit (innerhalb/zwischen) und die Beziehung zwischen Teams (Z) stärken könnte.

#### Assoziativ: Zugehörigkeit durch Territorialität

Teilnehmende berichteten, dass durch die räumliche Zusammenführung eines Teams (R 1) eine Art *territoriale Zuweisung *(Q 1) von Teammitgliedern entstand. Dies wurde von den Teilnehmern mit einem verstärkten *Teamzugehörigkeitsgefühl* (K 1) und einer gewissen *Angleichung sozialer Normen *(Rücksicht) (K 2) in Verbindung gebracht:

Du bist stärker verbunden mit den Leuten und du redest viel mehr und das Vertrauen hat sich durch das sicher auch gestärkt (Nr. 9).

Es gibt ein gutes Gefühl, weil man sich dann auch sehr nahe ist für den Austausch, wenn irgendetwas besprochen werden muss. Dass man da nicht irgendwie räumlich getrennt ist (Nr. 7).

Es ist (…) eine allgemeine Kultur entstanden, wann es sinnvoll ist, mal über den Tisch hinweg etwas zu klären oder wann man sich in ein Think Tank zurückzieht. Ich glaube das hat sich vor allem geändert (Nr. 4).

Was sich auch geändert hat, ist, dass man, (…) ein bisschen mehr Rücksicht nimmt. (…) man ist sich bewusst, wenn ich jetzt telefoniere, dann gehe ich in den Fokusraum. Das hat man früher direkt im Büro gemacht, da hat man nur einen gestört oder so, dann war das nicht so schlimm. Hier hört mans dann halt relativ schnell. Entsprechend zieht man sich dann zurück. (…) Ich glaube das hat sich auch geändert, dass man ein bisschen mehr aufpasst und oft ein bisschen mehr Rücksicht nimmt und dass man auch jemandem Rückmeldung gibt, wenns einem stört (Nr. 5)

Des Weiteren berichteten die Teilnehmenden, dass durch die räumliche Zusammenführung mehrerer Teams (R 2) eine Art *territoriale Teilung* (Q 2) und eine neue *Möglichkeit der Durchmischung* erleichtert. Einige assoziierten dies mit einer verstärkten *Offenheit, teamübergreifenden Zugehörigkeit* und/oder *Firmenidentifikation* (K 1–3):

Vorher (…) ein verschworenes Team (…) weil wir geographisch getrennt waren (…) jetzt (…) offener, weil (…) auf dem gleichen Geschoss mehrere Teams haben. (…) kann (…) sein, dass von einem anderen Team in dieser Zone jemand arbeitet (…) eine bessere Durchmischung von verschiedenen Teammitgliedern (Nr. 7).

Als wir dann hierhin kamen [zusammenführender Firmenstandort] mit all den anderen Leuten, hat die Zugehörigkeit sich stark verbessert. Und die Identifikation auch damit (…) Und wie gesagt, der übergreifende Zusammenhalt, da merkt man schon so ein bisschen der Spirit der Firma und das ist schon auch sehr cool. (Nr. 8).

Die Schilderungen der Teilnehmenden deuten darauf hin, dass eine territorial-räumliche Zusammenführung der Teams (Q 1–2), insgesamt das *Zugehörigkeitsgefühl in und zwischen Teams* (Z) fördern könnte.

### Arbeitsplatzgleichheit

#### Assoziativ: Kollegial-beratende Führungsbeziehung durch Symbolik und Territorialität

Einige Teilnehmende berichteten, dass die Angleichung der Arbeitsbedingungen (R 1) (Räumlichkeit und Ausstattung), eine *symbolische Angleichung des Status* (K 1) mit sich brachte, welche die psychologische Distanz (Q 1) zwischen Angestellten und Führungskräften verringerte. Ebenso schätzten sich Teilnehmende gleichbehandelt und gleichberechtigt (Nr. 9):

Es [war] wichtig zu kommunizieren, dass sie genauso einen Arbeitsplatz wie wir haben, wir arbeiten unter den gleichen Bedingungen, für alle gleich und das nimmt so ein bisschen wahrgenommene Distanz (Nr. 5).

Jeder ist bei uns gleichberechtigt. Auch mein Chef, auch mein Chef-Chef, hat auch auf sein Einzelbüro verzichtet und ist auch im Open Space integriert. Es werden wirklich alle Leute gleichbehandelt. Auch die Ausstattung ist die Gleiche. (…) es hat sich zum Positiven verändert. Die Hierarchiestufen haben sich (…) nicht verändert, aber man nimmt das Ganze viel flacher wahr (Nr. 9).

Des Weiteren scheinen die räumliche Zugehörigkeit (R 2) und die territoriale Teilung einer Zone (Q 2), die psychologische Distanz (Q 1) ebenso auf kohäsiver Ebene verringert zu haben. Teilnehmende erwähnten eine verstärkte *Präsenz der Führungskraft* im Team (K 1), ein verstärktes *Zugehörigkeitsgefühl *(K 2) und einen *transparenteren Umgang *(K 3):

Von da her spürt man sie mehr, oder man sieht sie mehr, diese Führungskräfte, weil sie halt auch in diesem Grossraumbüro sitzen (Nr. 6).

Vorher war der Chef irgendwo in einem Einzelbüro und da fragtest du dich: Darf ich den jetzt stören oder (…) wie ist er heute gelaunt? (…) viel transparenter, wenn die Leute wirklich auch im selben Raum sind oder neben dir sitzen sogar. Von daher ist es (…) besser als vorher (Nr. 9).

Weil ja der Abteilungsleiter auch in der gleichen Zone arbeitet. Das gibt schon ein Teamgeist (Nr. 7).

Sie [haben] eher eine Coachingfunktion (…) Du nimmst diese Leute eher als Teamkollegen wahr anstelle des Chefs. Das heisst nicht, dass wir keinen Respekt haben von diesen Leuten *lacht*, das ist schon klar. (…) die Nähe zum Chef wird gefördert. Der Draht wird ein wenig gestärkt. Das ist sicher sehr gut und wurde durch die [räumliche] Umstrukturierung gestärkt (Nr. 9).

Die Schilderungen der Teilnehmenden geben Anlass zu vermuten, dass eine symbolisierte Ebenbürtigkeit und Teamzugehörigkeit (Q 1, Q 2), eine *kollegial-beratende* und weniger formelle *Führungsbeziehung* (Z) fördern könnte.

## Diskussion

Die Studie verfolgte die Ziele, bestehende Erkenntnisse bezüglich des Zusammenhanges von ABW Designmerkmalen und Organisationskulturparametern in Hinblick auf Clan-Kulturen qualitativ zu überprüfen, aus anderen Studienkontexten zu übertragen, sowie neue Zusammenhänge zu identifizieren.

### Visueller Zugang und Clan-Kultur: Kommunikation Dynamik

Kulturveränderungen durch Schaffung von visuellem Zugang wurden *funktionalen* sowie *assoziativen* Mechanismen zugeordnet.

Berichtete *funktionale *Einflüsse wurden vornehmlich mit einer clan-konforme Veränderungen der *Kommunikation* hinsichtlich der Häufigkeit sowie Art des Austausches in Verbindung gebracht. Diese indikativen Zusammenhänge bestätigen bekannte Evidenzen zur gesteigerten Kommunikationshäufigkeit in und zwischen Teams, auch wenn diese Studien keinen Fokus auf Designelemente oder Organisationskultur legten (Allen und Gerstberger 1973; Becker und Sims 2001; Boutellier et al. 2008; Stryker 2004). Die wahrgenommene Veränderung in der Art der Kommunikation, die im Team *öfter, kürzer, spontaner, informeller *(ähnlich Becker und Sims 2001; Boutellier et al. 2008) und *fokussierter* und zwischen Teams *informeller* wurde (ähnlich De Been et al. 2015), ist mehrheitlich belegt. Zwar wurde bereits auf besseren Informationsfluss und schnelleres Fällen von Entscheidungen, aufgrund der Offenheit des Layouts hingewiesen (Becker und Sims 2001; Boutellier et al. 2008), allerdings wurde der fokussierte Austausch noch nicht explizit aufgeführt. Dieses Phänomen könnte mit dem theoretischen Ansatz der Informationsredundanz in geteilten Arbeitsterritorien (Nonaka 1990) erklärt werden.

*Assoziative Einflüsse *wurden vornehmlich mit einer *dynamische Atmosphäre *und dem *Gefühl der Zughörigkeit* in Verbindung gebracht, welches durch Sichtachsen und dem besseren Überblick über das Team begünstigt wurde. Die bis anhin in der Literatur erwähnte *dynamische Atmosphäre *wurde räumlich nicht näher differenziert, sondern ausschliesslich mit Offenheit des Layouts in Verbindung gesetzt (De Been et al. 2015); in der vorliegenden Studie wurde dieser Aspekt vor allem mit einer schnelleren und spontaneren Kommunikationsform in Zusammenhang gebracht und könnte einem hierarchie-flachen Verhalten der Clan-Kultur entsprechen (Cameron und Quinn 2011). Das verstärkte *Gefühl der Teamzugehörigkeit, *ein essentieller Bestandteil der Clan-Kultur (Hartnell et al. 2011), wurde mit verbessertem Überblick über das Team in assoziative Verbindung gebracht. Ein naheliegender Erklärungsansatz bietet die Territorialität. Durch den visuellen Zugang zum gesamten Team wird das geteilte Team-Territorium erfahrbar gemacht; symbolisierte Territorien können die Bindung zur zugehörigen Gruppe stärken (z. B. Altman 1975), auch in sekundären Territorien (Brown und Werner 1985), zu denen u. a. Arbeitsplätze zählen. Bisherige Studien berichteten indirekt von *Zugehörigkeitsgefühl* durch visuellen Zugang (Becker und Sims 2001; Skogland 2017), jedoch wurden andere Ausprägungen (verstärkte Rücksichtnahme) beobachtet und Mechanismen (verbesserter nicht-verbaler Kommunikation; Angleichung sozialer Normen) vermutet.

### Räumliche Nähe und Clan-Kultur: Zusammenarbeit Zugehörigkeit

Kulturveränderungen durch Schaffung von räumlicher Nähe wurden *funktionalen* sowie *assoziativen* Mechanismen zugeordnet. *Funktionale *Einflüsse wurden vornehmlich mit einer *verbesserten Zusammenarbeit in Teams* (Koordination, Informationsaustausch, Produktivität) sowie *teamübergreifende Zusammenarbeit und Beziehungen* in Verbindung gebracht; diese qualitativen Evidenzen decken sich mit bestehenden Evidenzen.

Wie bereits in ABW-ähnlichen (Becker und Sims 2001; Boutellier et al. 2008) und ABW-unspezifischen Studien (Allen und Gerstberger 1973; Hong et al. 2006; Kraut et al. 1990; Stryker 2004; Zahn 1991) berichtet wurde, kann räumliche Nähe die Zusammenarbeit fördern. In der vorliegenden Studie gibt es Hinweise, dass die räumliche Zusammenführung eines Teams in Zusammenhang mit einer Vereinfachung funktionaler Aspekte der Teamarbeit steht; Mitarbeitende wurden zugänglicher, was mit vereinfachter Koordination und Planung (wie Allen und Gerstberger 1973), schnellerem Informationsaustausch (wie Boutellier et al. 2008) und erhöhter Teamproduktivität (ähnlich Becker und Sims 2001) in Verbindung gebracht wurde. Des Weiteren gab es Hinweise darauf, dass zufällige Begegnungen (Serendipität), teil-orchestriert durch die räumliche Nähe anderer Teams, neue teamübergreifende Zusammenarbeit fördern kann (wie De Been et al. 2015; Kraut et al. 1990; vgl. Kiesler und Cummings 2002).

*Assoziative Veränderungen* durch die territorial-räumliche Zusammenführung eines und mehrerer Teams wurden vornehmlich in Verbindung gebracht mit *Zugehörigkeit, Identifikation mit Team und Unternehmen, Offenheit, *sowie* geteilte soziale Normen. *Diese qualitativen Erkenntnisse decken sich weitgehend mit bekannten Evidenzen. *Zugehörigkeit* und *Gruppenidentifikation* durch räumliche Nähe und territoriale Zuweisung ist mehrheitlich empirisch belegt (vgl. Kiesler und Cummings 2002; Elsbach und Bechky 2007); ein Aufbrechen von Silodenken, verstärkte Offenheit gegenüber anderen Teams und verstärkte Unternehmensidentifikation (zusätzlich zur Teamidentifikation) ist in den vorliegenden Studien nur indirekt festgehalten worden, jedoch nicht in vergleichbarer Ausprägung. Allen und Gerstberger (1973) befürchteten ein verstärktes Silodenken durch verstärkte Teamidentifikation, waren allerdings überrascht, einen kurzzeitigen Anstieg an teamübergreifendem Austausch festzustellen. Ebenso wiesen Kiesler und Cummings (2002) darauf hin, dass die Tendenz, lokale/Gruppen-Territorien zu etablieren, die kollektive Unternehmensidentifikation behindern kann. Eine Angleichung *sozialer Normen* wurden in vorherigen Studien vornehmlich mit visuellem Zugang in Verbindung gebracht (Skogland 2017; Becker und Sims 2001). Dies illustriert die geringe Differenzierungsmöglichkeit vorheriger Studien aufgrund der mangelnden Fokussierung auf räumliche Elemente.

### Arbeitsplatzgleichheit und Clan-Kultur: Führungskultur

Kulturveränderungen durch Arbeitsplatzgleichheit wurden *assoziative Mechanismen *zugeordnet. Es gab Hinweise, dass selbige eine *kollegial-beratende Führungskultur*, gemäss Clan-Kultur (Cameron und Quinn 2011), fördern könnte. Das *Angleichen der Arbeitsplatzbedingungen* wurde mit einer gefühlten *Angleichung des Status *zwischen Angestellten und Führungskräften in Verbindung gebracht. Des Weiteren gab es Hinweise, dass durch die *territoriale Teilung* in einer Arbeitszone Führungskräfte verstärkt als Teil des Teams wahrgenommen wurden und dies einen transparenteren Umgang über Hierarchiestufen hinweg fördern könnte (Becker und Sims 2001). Durch das *Angleichen der Arbeitsplatzbedingunge*n und die *territoriale Teilung *scheint sich die *psychologische *Distanz zwischen den Hierarchiestufen (Becker und Sims 2001) verringert zu haben. Einige Studien zeigten bereits auf, dass Arbeitsplatzunterschiede Status- und Machtunterschiede unterstreichen können (Zalesny und Farace 1987; Zhang und Spicer 2014). Die vorliegende Studie ergänzt diese Erkenntnisse, da sie sich auf die Angleichung von Arbeitsplatzbedingungen und nicht auf deren Unterschiede fokussiert.

Zusammenfassend lässt sich festhalten, dass eine Vielzahl bekannter Zusammenhänge anderer Studienkontexte auch im Studienkontext ABW-Design und Clan-Kultur-Indikatoren (*Kommunikation, Zusammenarbeit, Beziehungen, Zugehörigkeit* und *kollegial-beratende Führungskultur*) erscheinen. Des Weiteren gibt es Hinweise darauf, dass bekannte Zusammenhänge früherer Studien (Zerella et al. 2017) qualitativ bestätigt werden konnten. Neue identifizierte Themen oder Differenzierungen betreffen den*fokussierten Austausch, dynamische Atmosphäre, Offenheit* gegenüber anderen Teams und *Unternehmensidentifikation*. Als Erklärungsansätze und Einflussmechanismen dienen Funktionalität (Zugänglichkeit, Serendipität) und Assoziationen (Psychologische Distanz, Territorialität).

## Limitationen

Die Arbeit hat sechs Limitationen: Stichprobenart, Stichprobengrösse, Überprüfung des Einschlusskriteriums Arbeitskultur, Pandemieentwicklung, ausstehende Datentriangulation und kommunikative Validierung.

Die willkürliche Stichprobe sowie die geringe Stichprobengrösse birgt hinsichtlich der Komplexität der Forschungsfrage, trotz empfundener Datensaturierung, die Gefahr einer verringerten Reliabilität und Validität der Ergebnisse (z. B., Vasileiou et al. 2018).

Die Überprüfung des Einschlusskriteriums Clan-Kultur in Organisation oder Arbeitsgruppe erfolgte nicht über ein Messinstrument (z. B., Organizational Climate Measure (OCM), Patterson et al. 2005), sondern erfolgte mittels Selbsteinschätzung und Validierung durch die Forschungsgruppe.

Die einschneidenden Pandemie-Schutzmassnahmen im März 2020 könnten bei den im Home-Office (HO) interviewten Teilnehmenden zu einem gewissen Mass an Verzerrung geführt haben. Zum Bespiel könnten einige Themen unter Umständen in der HO- bzw. Pandemie-Situation salienter sein. Jedoch ist der verzerrende Effekt eher gering einzuschätzen, da die Interviewpartner erst seit kurz vor Interviewdurchführung hauptsächlich im HO arbeiteten; ebenso wurden in den Transkripten keine Hinweise auf besondere Salienz gewisser Themen gefunden. Des Weiteren könnten die zusätzlichen Herausforderungen des situationsbedingt digitaleren Arbeitsalltages zu einer geringeren Bereitschaft geführt haben, sich auf den Interviewprozess einzulassen. Allerdings wurden in den Notizen des Interviewers keine Anmerkungen dazu gemacht und die Interviews waren ähnlich in Länge (*M*_analog_ = 28 min vs. *M*_online_ = 24 min) und Inhaltsdichte.

Zwar erfolgte in der Analysephase eine Triangulation innerhalb der Forschungsgruppe, jedoch wurde keine Datentriangulation durch den Einsatz weiterer Methoden durchgeführt; insofern besteht das Risiko einer methodischen Verzerrung und verringerten Inhaltsvalidität (Steinke 2000).

Eine kommunikative Validierung oder „Member-Check“ der Ergebnisse durch die Interviewteilnehmer wurde ebenfalls nicht durchgeführt, was die interne Validität der Ergebnisse verringert (Steinke 2000).

Zukünftige Studien sollten die identifizierten assoziativen sowie funktionalen Zusammenhänge zwischen Designmerkmalen und Kultureigenschaften mittels grossflächigen qualitativen Studien prüfen. Trotz der positiven Darstellung des ABWs und assoziierten Clan-Kultur, ist es ausschlaggebend, dass dieses Bürokonzept prinzipienkonform umgesetzt wird und nicht nur Kommunikation und Zusammenarbeit fördert, sondern auch ausreichend Raum für individuelles und hochkonzentriertes Arbeiten und Rückzug bietet (z. B., Becker et al. 2021; Weber und Gatersleben 2021). Eine prinzipienkonforme ABW-Umsetzung generiert ein präferenzorientiertes Arbeitsplatzkonzept (Windlinger et al. 2014, 2015). Dieses bietet die Möglichkeit, sowohl den Zielen der Organisation und des Teams als auch den Bedürfnissen des Individuums gerecht zu werden (Becker et al. 2021; Parker 2016; Wohlers und Hertel 2018; Wohlers et al. 2019).

## Fazit

Die Studie gibt Hinweise darauf, dass ABW-typische Designmerkmale die Entwicklung einer Clan-Organisationskultur unterstützen können. Diese Erkenntnisse sind relevant für Forschung und Praxis, da wenige Studien Designmerkmale isoliert von anderen Eigenschaften der physischen Umgebung betrachtet haben. Die Erkenntnisse können einen Grundstein für vergleichende Untersuchungen von Designmerkmalen unterschiedlicher Ausstattungen legen, sowie Designstrategien unterstützen.

## Footnotes

1 Aufgrund mangelnder Differenzierung kann vorhergehenden Studien nur bedingt entnommen werden, ob untersuchte Einflüsse visuellem Zugang oder räumlicher Nähe zuzuschreiben sind. Evidenzen von Zerella et al. (2017) werden nicht aufgelistet, da korrelative Zusammenhänge nicht differenziert nach Kulturindikatoren analysiert wurden.

2 Vorherige Studien berichten von Assoziationen zwischen Gestaltungsqualitäten des Büros und wahrgenommener Wertschätzung der Organisation gegenüber den Mitarbeitenden (Windlinger et al. 2015; Elsbach und Bechky 2007).

## References

[CR1] Allen TJ, Gerstberger PJ (1973). A field experiment to improve communication in a product engineering department: the non-territorial office. Human Factors.

[CR2] Altman I (1975). The environment and social behavior: privacy, personal space, territory, crowding.

[CR4] Becker F, Sims W (2001). Offices that work: Balancing communication, flexibility and cost.

[CR3] Becker C, Soucek R, Gunkel J, Lütke Lanfer S, Göritz AS (2021). Tagebuchstudie zu Activity-Based Flexible Offices. Zeitschrift für Arbeits- und Organisationspsychologie AO.

[CR8] Boutellier R, Ullman F, Schreiber J, Naef R (2008). Impact of office layout on communication in a science-driven business. RD Management.

[CR5] Braun V, Clarke V (2006). Using thematic analysis in psychology. Qualitative Research in Psychology.

[CR6] Brill M, Weidemann S, Associates BOSTI (2001). Disproving widespread myths about workplace design.

[CR7] Brown BB, Werner CM (1985). Social cohesiveness, territoriality, and holiday decorations: the influence of cul-de-sacs. Environment and Behavior.

[CR9] Cameron KS, Quinn RE (2011). Diagnosing and changing organizational culture: based on the competing values framework.

[CR10] Crabtree B, Miller W, Crabtree B, Miller W (1999). A template approach to text analysis: developing and using codebooks. Doing qualitative research.

[CR11] De Been, I., Beijer, M., Den Hollander, D. (2015). How to cope with dilemmas in activity based work environments: results from user-centred research. *EuroFM Research Papers*, *14*, 1–10. https://www.cfpb.nl/kennis/publicaties/how-to-cope-with-dilemmas-in-activity-based-work-environments/. Zugegriffen: Januar 2020.

[CR12] Denison DR, Haaland S, Goelzer P (2004). Corporate culture and organizational effectiveness: is Asia different from the rest of the world?. Organizational Dynamics.

[CR13] Elsbach KD, Bechky BA (2007). It’s more than a desk: Working smarter through leveraged office design. California Management Review.

[CR14] Fereday J, Muir-Cochrane E (2006). Demonstrating rigor using thematic analysis: a hybrid approach of inductive and deductive coding and theme development. International Journal of Qualitative Methods.

[CR15] Fuchs B, Kuk T, Wiechmann D, Seiferlein W, Kohlert C (2018). Adäquate Büroeinrichtung. Die vernetzten gesundheitsrelevanten Faktoren für Bürogebäude. Geplante Gesundheit.

[CR16] Fusch PI, Ness LR (2015). Are we there yet? Data saturation in qualitative research. The Qualitative Report.

[CR17] Hartnell CA, Ou AY, Kinicki A (2011). Organizational culture and organizational effectiveness: a meta-analytic investigation of the competing values framework’s theoretical suppositions. Journal of Applied Psychology.

[CR18] Hirst A (2011). Settlers, vagrants and mutual indifference: Unintended consequences of hot-desking. Journal of Organizational Change Management.

[CR19] Hong JFL, Easterby-Smith M, Snell RS (2006). Transferring organizational learning systems to Japanese subsidiaries in China. Journal of Management Studies.

[CR20] Howard LW (1998). Validating the competing values model as a representation of organizational culture. The International Journal of Organizational Analysis.

[CR21] Kiesler S, Cummings J, Hinds PJ, Kielser S (2002). What do we know about proximity and distance in work groups? A legacy of research. Distributed Work.

[CR2015] Klaffke, M., Kuchta, A. (2015). Zu Besuch im Büro der Zukunft. *Personalmagazin* 09/2015 (S. 34–36).

[CR22] Kraut RE, Fish RS, Root RW, Chalfonte BL, Oskamp IS, Spacapan S (1990). Informal communication in organizations: form, function, and technology. Human reactions to technology: the claremont symposium on applies social psychology.

[CR23] McElroy JC, Morrow PC (2010). Employee reactions to office redesign: a naturally occurring quasi-field experiment in a multi-generational setting. Human Relations.

[CR24] Nanayakkara K, Wilkinson S, Appel-Meulenbroek R, Danviska V (2021). Dimensions of organisational culture and office layouts. A handbook of theories on designing fit between people and the office environment.

[CR25] Nonaka I (1990). Redundant, overlapping organization: a Japanese approach to managing the innovation process. California Management Review.

[CR26] Parker LD (2016). From scientific to activity based office management: a mirage of change. Journal of Accounting Organizational Change.

[CR27] Patterson MG, West MA, Shackleton VJ, Dawson JF, Lawthom R, Maitlis S (2005). Validating the organizational climate measure: links to managerial practices, productivity and innovation. Journal of Organizational Behavior.

[CR28] Schein EH (2004). Organizational culture and leadership.

[CR29] Skogland MAC (2017). The mindset of activity-based working. Journal of Facilities Management.

[CR30] Steinke I, Flick U, von Kardorff E, Steinke I (2000). Gütekriterien qualitativer Forschung. Qualitative Forschung. Ein Handbuch.

[CR31] Stryker JB (2004). Designing the workplace to promote communication: the effect of collaboration opportunity on face-to-face communication in RD project teams.

[CR32] Vasileiou K, Barnett J, Thorpe S, Young T (2018). Characterising and justifying sample size sufficiency in interview-based studies: systematic analysis of qualitative health research over a 15-year period. BMC Medical Research Methodology.

[CR33] Vilnai-Yavetz I, Rafaeli A, Yaacov CS (2005). Instrumentality, aesthetics, and symbolism of office design. Environment and Behavior.

[CR34] Weber C, Gatersleben B (2021). Office relocation: changes in privacy fit, satisfaction, and fatigue. Journal of Corporate Real Estate.

[CR35] Windlinger, L., Gersberg, N., Konkol, J. (2015). Unterstützung mobil-flexibler Arbeit durch aktivitätsorientierte Gestaltung von Büroräumen. *Wirtschaftspsychologie, 16*(4), 83–95. https://digitalcollection.zhaw.ch/handle/11475/6368. Zugegriffen: Dezember 2019.

[CR36] Windlinger, L., Konkol, J., Schanné, F., Sesboüé, S., Neck, R. (2014). Gesundheitsförderliche Büroräume. https://gesundheitsfoerderung.ch/assets/public/documents/de/5-grundlagen/publikationen/bgm/berichte/Bericht_004_GFCH_2014-06_-_Gesundheitsfoerderliche_Bueroraeume.pdf. Zugegriffen: Januar 2020.

[CR38] Wohlers C, Hertel G (2018). Longitudinal effects of activity-based flexible office design on teamwork. Frontiers of Psychology.

[CR37] Wohlers C, Hartner-Tiefenthaler M, Hertel G (2019). The relation between activity-based work environments and office workers’ job attitudes and vitality. Environment and Behavior.

[CR39] Zahn GL (1991). Face-to-face communication in an office setting: the effects of position, proximity, and exposure. Communication Research.

[CR40] Zalesny MD, Farace RV (1987). Traditional versus open offices: a comparison of sociotechnical, social relations, and symbolic meaning perspectives. Academy of Management Journal.

[CR41] Zerella S, von Treuer K, Albrecht SL (2017). The influence of office layout features on employee perception of organizational culture. Journal of Environmental Psychology.

[CR42] Zhang Z, Spicer A (2014). “Leader, you first”: the everyday production of hierarchical space in a Chinese bureaucracy. Human Relations.

